# Focus on Basal Cell Carcinoma

**DOI:** 10.1155/2011/328615

**Published:** 2010-10-24

**Authors:** Venura Samarasinghe, Vishal Madan, John T. Lear

**Affiliations:** ^1^Manchester Royal Infirmary, Central Manchester and Manchester Childrens' Hospital NHS Trust, Oxford Road, Manchester M13 9WL, UK; ^2^The Dermatology Centre, Salford Royal Hospital NHS Foundation Trust, Stott Lane, Salford M6 8HD, UK

## Abstract

Nonmelanoma skin cancers (NMSCs), which include basal and squamous cell cancers are the most common human cancers. BCCs have a relatively low metastatic rate and slow growth and are frequently underreported. Whilst there is a definite role of sunexposure in the pathogenesis of BCC, several additional complex genotypic, phenotypic and environmental factors are contributory. The high prevalence and the frequent occurrence of multiple primary BCC in affected individuals make them an important public health problem. This has led to a substantial increase in search for newer noninvasive treatments for BCC. Surgical excision with predetermined margins remains the mainstay treatment for most BCC. Of the newer non-invasive treatments only photodynamic therapy and topical imiquimod have become established in the treatment of certain BCC subtypes, while the search for other more effective and tissue salvaging therapies continues. This paper focuses on the pathogenesis and management of BCC.

## 1. Introduction

Basal cell carcinoma (BCC) is the commonest skin cancer in Caucasians and its incidence continues to increase worldwide [1]. First described in 1824 by Jacob, it is a slow growing, locally destructive, skin tumour of the epidermis [2]. Average life time risk of developing a BCC is approximately 30% in Caucasians, and represents a significant public health issue [3]. Australia has the highest incidence of BCC in world with 1383 new cases diagnosed for every 100,000 population in 2008 [4]. In UK there are estimated 53,000 new cases per year, and 153.9 cases per 100, 000 person years [5]. Figures for incidence are likely to be significantly underestimated in the UK as BCCs are not routinely registered. 

 Fortunately in respect of the high incidence, BCCs rarely metastasise. Metastatic rate is <0.1%, and reported sites of metastasis are skeletal and pulmonary [6, 7]. Risk factors for metastasis are neglect over many years, perineural invasion, size over 10 cm^2^, and basisquamous and morphoeic subtypes [8]. Tumours left untreated can cause extensive tissue destruction, disfigurement, infiltrate cartilage, muscle or bone with even intracranial extension. BCCs may also develop in scars or sebaceous naevi and are associated with several genetic syndromes including basal cell naevus (Gorlin's) syndrome, xeroderma pigmentosa, Bazex syndrome, and albinism [9]. 

There are several subtypes of BCC: nodular, cystic, superficial, morphoeic (sclerosing), keratotic, pigmented, and micronodular [10]. Nodular BCCs are the most common in UK, though 10–40% show a mixed pattern of 2 or more subtypes [11]. Most nodular and morphoeic subtypes are found on the head and neck, contrasting with 46% of superficial BCC occurring over truncal sites [12].

Once a BCC is diagnosed, risk of further BCC is approximately 10-fold over the general population. Risk factors for developing further BCCs are multiple BCC at presentation, older age at presentation, and index BCC over 1 cm. BCC arising on the trunk are usually associated with development of further truncal BCC [10]. Overall risk of further BCC is approximately 46% over a 10-year period [13]. 

## 2. Aetiology

Aetiology of BCC is a multifactorial combination of genotype, phenotype, and environmental factors. Ultraviolet radiation is one the most significant factors, demonstrated by the global highest incidence in areas closer to the equator, whilst Finland has the lowest incidence of all European countries. UVB irradiation directly damages cellular DNA and RNA causing covalent bonding between adjacent pyrimidines, and formation of mutagenic products. UVA produces the formation of toxic reactive oxygen species [14]. Overall intermittent sun exposure and childhood sun exposure may be of more importance than cumulative sun exposure [15, 16].

Several genes have been associated with BCC development. Cytochrome 450 (CYP) and glutathione S-transferase (GST) are both involved in detoxifying various mutagens. Specific polymorphisms within these supergenes have been identified, in particular GSTM1, GSTT1, GSTP1 and CYP2D6 [17, 18]. CYP2D6 may be associated with the development of multiple BCCs also [19]. Basal cell naevus syndrome (BCNS) is due to mutation in the PTCH gene located on chromosome 9q22. PTCH gene is the human homologue of the drosophilia patched gene, negatively regulating Hedgehog signalling via inhibition of Smoothened (Smo), a transmembrane protein. Inactivating mutations of PTCH in BCNS stimulate aberrant Hedgehog signalling and subsequent BCC development. Sporadic BCCs have also been shown to contain PTCH mutations in up to 68% of cases. Mutations in tumour suppressor gene p53 cause inactivation of the gene and development of tumours resistant to apoptosis. Up to 53% of BCCs may have a single allele mutation of p53 [20]. Skin type is associated with melanocortin 1 receptor (MC1R) polymorphisms and an independent risk factor for BCC. Eye colour and tanning function is associated with polymorphisms in tyrosinase and subsequent risk of BCC development [21].

Other important risk factors are age over 40, sun bed use, phototherapy, radiotherapy, male sex, and arsenic exposure [10]. Immunosuppression confers a 10–100 increased risk over the general population, a risk which increases with longer duration of immunosuppression. Fair skin types (Fitzpatrick skin type I and II) are also at increased risk of developing BCC, with 19 times reduced risk in darker skin [14]. 

## 3. Diagnosis

**Figure 1:**
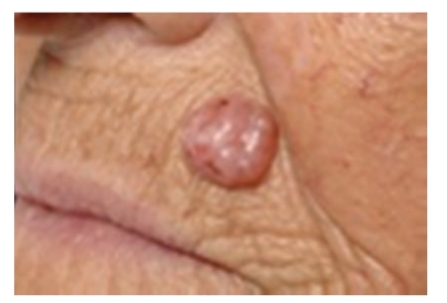
Nodulo-cystic BCC.

**Figure 2:**
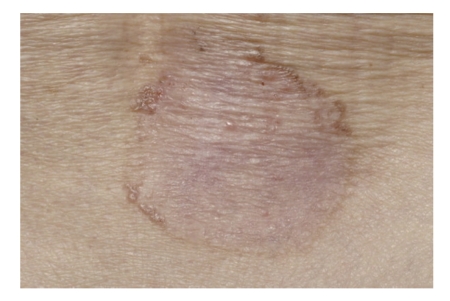
Superficial BCC.

**Figure 3:**
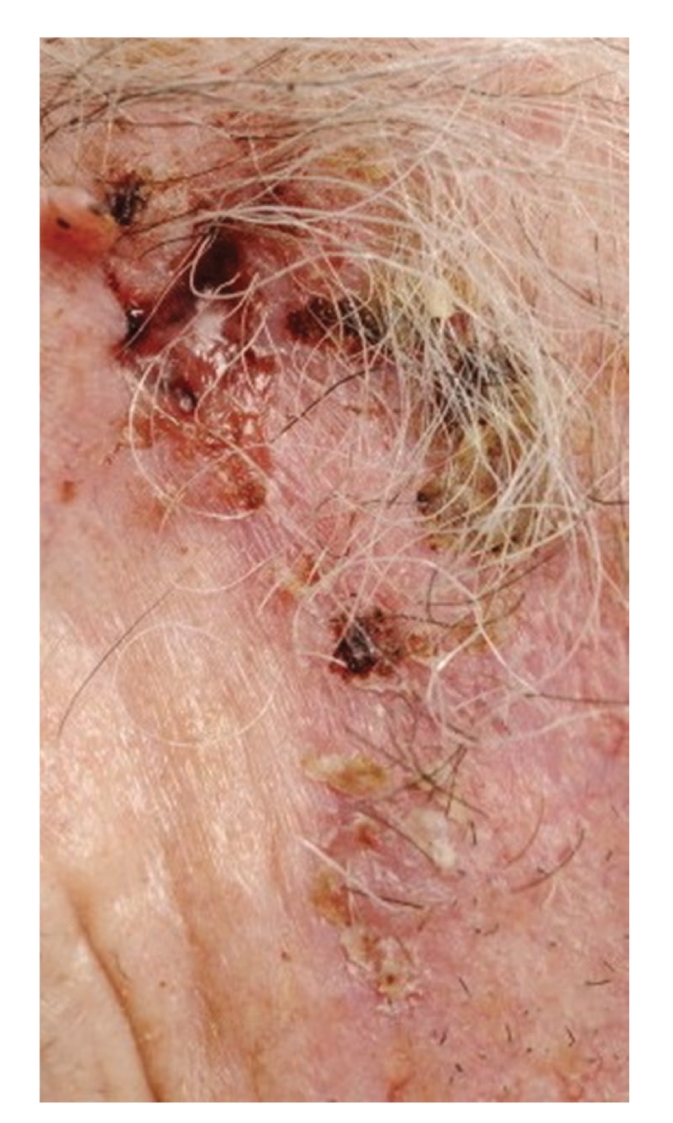
Morphoeic BCC.

 Diagnosis is usually clinical. Clinical features are dependent on the subtype of BCC. Nodular or cystic BCCs present as raised red, pearly, translucent lesions with peripheral telangiectasia (Figure 1). Superficial BCC may mimic discoid eczema or Bowen's disease whilst morphoeic BCC presents as a subtle scar-like plaque (Figures 2 and 3). Dermatoscopy may be helpful to identify arborising blood vessels, ulceration, maple-leaf-like areas characteristic of BCC [22]. Computer tomography or magnetic resonance imaging is performed for bony, vascular, or major nerve invasion. Skin biopsy is performed in the majority of cases to aid diagnosis and identify subtype of BCC for treatment planning.

## 4. Treatment

Treatment of BCC is hampered by poor quality, conflicting research with often short-term 1 year clearance data. Most studies are open, uncontrolled, and retrospective in nature. The key decision for treatment is identifying high versus low risk BCC (Table 1). Standard surgical excision and Moh's micrographic surgery allows histological confirmation of tumour clearance and generally remains gold standard for high risk BCC, whilst the other treatment modalities rely on clinical observation at followup to confirm treatment success, with higher recurrence rate at 5-year review.

## 5. Surgical Excision

The BCC is excised with a predetermined margin of usually 3-4 mm of normal skin to ensure the lesion is fully excised. The excised tissue is placed in formalin, embedded, and cut into interrupted vertical sections akin to a “breadloaf” for histological review. As the entire margin is not examined, this allows for sampling error and reporting of BCC to be completely excised when in fact they may not be. In 2004 a prospective randomised controlled study of surgical excision versus Mohs' micrographic surgery by Smeets et al., found of 199 primary facial BCC excised with 3 mm margins, 18% were incompletely excised at first attempt, requiring further excision [23]. Tumours which were of aggressive histological subtype were significantly more likely to be incompletely excised initially. 14% had complications, most commonly wound infection, or necrosis of graft or flap rather than bleeding or haematoma. Overall recurrence rate of BCC with surgical excision was 4.1% at 5 year followup. A larger retrospective review by Rowe et al., of all studies published since 1947 found a higher recurrence rate of 10.1% at 5-year followup for standard surgical excision [24].

## 6. Moh's Micrographic Surgery

Moh's micrographic surgery (MMS) was first reported in 1941 by Mohs [25]. Excised tissue is frozen and sectioned horizontally. The entire margin is intraoperatively histologically examined and further staged excision is performed only where the tumour is located microscopically. This allows for greater histological accuracy of complete tumour resection and increased tissue conservation. In the study by Smeets et al., comparing surgical excision versus Moh's micrographic surgery, 198 patients were randomised to the MMS arm. Complication rate for MMS was 12%, most commonly wound infection and flap/ graft necrosis. 5-year recurrence rate was 2.1%, which was not significantly different in comparison to surgical excision (*P* = .23). Neither was a significant difference found in patient perception of cosmetic appearance between MMS and surgical excision for primary BCC.

However for recurrent facial BCC, MMS is the treatment of choice. 204 patients with recurrent facial BCC were also randomised to MMS versus surgical excision. At 5-years 2.4% recurred in the MMS group in comparison to 12.1% in the surgical group, confirming significantly lower recurrence in the MMS group (*P* = .015). In addition 30% of tumours in surgical group were initially incompletely excised and required further surgery [23]. Similar results were found in Rowe et al.'s retrospective review of all studies since 1945, with 1.0% 5-year recurrence for untreated BCC with MMS and 5.6% 5 year recurrence rates for treatment of recurrent BCC with MMS [24, 26]. 

## 7. Radiotherapy

Radiotherapy (RT) can be effective for primary BCC, recurrent BCC or as adjuvant for incompletely excised BCC in patients where further surgery is neither possible nor appropriate. Radiotherapy is a mixture of superficial, electron beam, and brachytherapy for curved surfaces. Treatment in fractions over several visits may produce better cosmetic outcomes than a single fraction treatment [10]. Radiotherapy is contraindicated in radiotherapy recurrent BCC, genetic syndromes predisposing to skin cancer and connective tissue disease. Significant side effects are radionecrosis, atrophy, and telangiectasia. Skin cancers can arise from radiotherapy field scars and should be avoided in younger age groups.

A randomised trial of 347 patients compared radiotherapy versus surgical excision with frozen sections for facial BCC less than 4 cm found significantly more recurrence occurred in the radiotherapy group (7.3%) than the surgical group (0.7%) at 4-year followup (RR 0.09, 95% CI, 0.01 to 0.69). Cosmetic outcome also significantly favoured surgical excision at 4 years with 87% of patients assessing the surgical scar as good, compared to 69% after radiotherapy (*P* < .01) [27, 28].

## 8. Curettage and Cautery

Curettage and cautery is one of the commonest tools used by dermatologists in management of BCC. The tumour is scraped with a curette and then treated with electrocautery to control bleeding and destroy any cancer cells at the base and margin of the wound. The cycle is repeated either once or twice for increased efficacy. A study of 898 BCC reported a 5 year cumulative recurrence rate of 6–19%. Recurrence was higher for central facial areas [29]. A further study of 2314 BCC treated with curettage and cautery also confirmed significantly higher recurrence for facial BCCs [30].

## 9. Cryotherapy

Cryotherapy is a destructive method of treating BCC using liquid nitrogen to cause low cell temperature and necrosis. Significant variation occurs in technique, length, and number of freeze thaw cycles used. Side-effects include pain, blistering, infection, and scarring. A nonrandomised study of 93 patients comparing cryotherapy to radiotherapy, treated BCC with 2 freeze thaw cycles of freezing for 1 minute, and thaw of 90 seconds. At 2-year followup, in the cryotherapy group 39% of BCCs had recurred, compared to 4% in the radiotherapy group. No significant difference was found in the cosmetic outcome between cryotherapy and radiotherapy [31]. Another study compared cryotherapy to surgical excision for head and neck superficial and nodular BCC, less than 2 cm. Cryotherapy was performed using 2 freeze thaw cycles of freezing for 20 seconds, and thaw of 60 seconds. No difference in recurrence rate was found at 1 year, but there was a significantly better cosmetic outcome with surgical excision compared to cryotherapy [32].

## 10. Photodynamic Therapy

Photodynamic therapy (PDT) is performed by topical application of the prodrug 5-aminolaevulinic acid (ALA) or methyl aminolaevulinic (MAL) to the BCC. The prodrug is converted intracellularly into protoporphyrin IX (PpIX) via the heme pathway. The BCC is then irradiated with a light source and in the presence of oxygen, a cytotoxic reaction occurs within the target cells where the PpIX has accumulated. The light source is usually either 410 nm blue light or 630 nm red light to match the absorption peak for PpIX. Red light may be preferred with the lipophilic MAL for deeper tissue penetration. Superficial BCC has been shown to achieve 87% clearance, and better cosmesis than with curettage or cryotherapy [33].

For nodular BCC a study comparing MAL PDT with surgical excision in 101 patients showed a MAL PDT cure rate of 76% compared to 96% for surgical excision. Cosmesis was better for PDT with 87% of patients rated as good cosmetic outcome in comparison to 54% for surgery [34].

## 11. Imiquimod

Imiquimod is an immune response modifier, binding to cell surface toll receptor 7 and/or 8. Binding activates proinflammatory cytokine production and subsequent cytotoxic T cell mediated cell death. Studies of topical administration for low risk BCC at least 5 days per week confirmed 81% histological clearance at week 6 or 12 [35–37]. A further study of topical imiquimod applied 5 days per week for a total of 6 week in 182 patients found 69% remained clinically clear at a 5 year followup period [38]. Side effects included application site erythema, crusting, erosion, and pain.

## 12. Conclusion

Research has improved our understanding of the pathogenesis of basal cell carcinoma, and with this has arrived several new generation nonsurgical treatments. However Moh's micrographic surgery remains gold standard for high risk BCC. Choice of treatment for basal cell carcinoma is complex and must take into account tumour type, location, cosmesis, recurrence, comorbidity and patient preference.

## Figures and Tables

**Table 1:** Factors associated with high risk of future BCC recurrence.

High Risk BCC
Aggressive histological subtype; morphoeic, micronodular
Large size >2 cm
Perineural, perivascular invasion
Central facial site; periocular, nasal, perioral
Recurrent BCC
